# The potential influence and intervention measures of gut microbiota on sperm: it is time to focus on testis-gut microbiota axis

**DOI:** 10.3389/fmicb.2024.1478082

**Published:** 2024-10-08

**Authors:** Wenkang Chen, Hede Zou, Haoran Xu, Rui Cao, Hekun Zhang, Yapeng Zhang, Jiayou Zhao

**Affiliations:** ^1^Graduate School of China Academy of Chinese Medical Sciences, Beijing, China; ^2^Graduate School of Hebei University of Chinese Medicine, Shijiazhuang, China

**Keywords:** gut microbiota, spermatogenesis, sperm motility, testis-gut microbiota axis, dual impact, mechanism, intervention measures

## Abstract

As the global male infertility rate continues to rise, there is an urgent imperative to investigate the underlying causes of sustained deterioration in sperm quality. The gut microbiota emerges as a pivotal factor in host health regulation, with mounting evidence highlighting its dual influence on semen. This review underscores the interplay between the Testis-Gut microbiota axis and its consequential effects on sperm. Potential mechanisms driving the dual impact of gut microbiota on sperm encompass immune modulation, inflammatory responses mediated by endotoxins, oxidative stress, antioxidant defenses, gut microbiota-derived metabolites, epigenetic modifications, regulatory sex hormone signaling. Interventions such as probiotics, prebiotics, synbiotics, fecal microbiota transplantation, and Traditional natural herbal extracts are hypothesized to rectify dysbiosis, offering avenues to modulate gut microbiota and enhance Spermatogenesis and motility. Future investigations should delve into elucidating the mechanisms and foundational principles governing the interaction between gut microbiota and sperm within the Testis-Gut microbiota Axis. Understanding and modulating the Testis-Gut microbiota Axis may yield novel therapeutic strategies to enhance male fertility and combat the global decline in sperm quality.

## 1 Introduction

Infertility is becoming a widespread global issue, and the World Health Organization estimates that 12.6–17.5% of couples globally encounter fertility challenges, with male factors contributing to 30–50% of cases of reduced fertility (Cox et al., 2022; Fainberg and Kashanian, 2019; Eisenberg et al., 2023), causing social, psychological, and marital problems for couples. Male reproductive impairment can arise from factors impacting sperm production, quality, function, or transport (Tournaye et al., 2017). Given that male fertility hinges on both sperm quantity and quality, semen quality serves as a crucial indicator of male reproductive health and is closely linked to fertility. A retrospective analysis of semen samples collected globally throughout the 20th and 21st centuries has revealed a significant decline in male sperm concentration and total sperm count, a trend that is accelerating in the 21st century (Levine et al., 2023). With the acceleration of global male infertility, there is an urgent need to investigate the potential causes and mechanisms of this continuous decline, and take preventive measures to protect male reproductive health from further deterioration.

The gut microbiota (GM) represents a significant component of the gastrointestinal tract, often termed the “second human genome” due to its vast repertoire of over 3 million genes, compared to the approximately 23,000 genes found in the human genome. It is recognized as the host’s endocrine organ. This extensive bacterial community plays a critical role in preserving the equilibrium between the host’s internal and external environments, thereby serving as a pivotal determinant of host health (De Vos et al., 2022; Lloyd-Price et al., 2016). The GM constitutes a complex and dynamically changing microbial community. Across the life cycle of mammals, evolution has occurred in conjunction with this microbiota (Argaw-Denboba et al., 2024), and mounting research underscores the pivotal role of GM in human physiological functions and disease progression. The indigenous GM fulfills distinct functions in host nutrient metabolism, xenobiotic and drug metabolism, preservation of the structural integrity of the gut mucosal barrier, immunomodulation, and defense against pathogens (Jandhyala et al., 2015). Research indicates that nearly all regions of the human body harbor microorganisms, and various organs can communicate through the GM (Gilbert et al., 2018; Schmidt et al., 2018). Furthermore, different individuals can also share connections via their GM. A recent study (Argaw-Denboba et al., 2024) highlights the pivotal role of the GM in mediating intergenerational health outcomes across paternal lineages in mice. Disruption in the ecological balance of the paternal GM has been linked to alterations in the male reproductive system, including compromised leptin signaling, changes in testicular metabolite profiles, and the redistribution of small RNA payloads in sperm. These changes increase the risk of developmental disorders and premature mortality in offspring, directly impacting their overall health.

Recent studies have demonstrated the significant influence of GM on sperm. Increasing research indicates an interplay between GM and the male reproductive system, highlighting its pivotal role in reproductive health (Hao et al., 2022; Yan et al., 2022; Jin et al., 2024; Li et al., 2022c; Li et al., 2024). Numerous studies have examined how GM impacts semen from dual perspectives (Ding et al., 2020; Lundy et al., 2021). On one hand, GM like Lactic acid bacteria, Bacteroidetes, and Ruminococcus (UCG011) can enhance sperm production, motility, and semen quality. (Fu et al., 2023). On the other hand, imbalanced GM can disrupt sperm production and reduce motility. The negative correlation between sperm motility and some “bad bacteria” or GM dysbiosis has been identified, for example Bacteroidetes Prevotella, *Enterococcus faecalis*, and GM dysbiosis caused by high-fat diet or otheres. The potential mechanism of GM influencing on sperm includes GM metabolites or bacterial cells regulating host intestinal homeostasis, host metabolism, that finally affects host reproductive function (Fu et al., 2023; Lv et al., 2024).

The influence of GM on sperm is evident, however, the specific influencing mechanisms require further elucidation. Accordingly, our team maintains a focus on investigating the gut-testis axis (Zou et al., 2024). By thoroughly reviewing existing literature, we aim to uncover potential mechanisms through which GM affects semen. This exploration intents to stimulate researchers’ interest on investigating the connections and reciprocal influences between GM and reproductive disorders, including sperm health, testicular function, and sex hormone regulation.

## 2 Potential mechanisms of GM affecting sperm

The GM comprises numerous species and interacts with multiple systems in the body, exhibiting complex potential mechanisms of action (Chen et al., 2021). This section focuses on elucidating the dual effects of GM on semen, exploring its role in mediating immune and inflammatory responses via endotoxins, oxidative stress, antioxidant protection, microbiota-derived metabolites, epigenetic modifications, regulation of sex hormones, and modulation of the blood-testis barrier.

### 2.1 GM mediates immune and inflammatory responses via endotoxins

Endotoxin is a potential pathway through which the intestinal microbiota mediates immune and inflammatory responses that affect sperm generation and reproductive function (Noguchi et al., 2017; Tremellen et al., 2018; Khanmohammad et al., 2021). Endotoxin is a component of the intestinal microbiota, particularly gram-negative bacteria, which use lipopolysaccharides (LPS) as cytoderm, that is effective activator of inflammation. Upon activation of the immune system, inflammatory mediators such as cytokines (e.g., tumor necrosis factor, interleukin-6) and chemokines (Chen et al., 2017; Silva et al., 2018) are typically released, triggering inflammatory responses that can affect sperm generation and function (Rizzetto et al., 2018; Maynard et al., 2012; Brown et al., 2019; Wei et al., 2024).

Dysbiosis of the GM can lead to the release of endotoxins into the intestine due to damage of gram-negative bacteria (Zhao et al., 2019; Schoeler and Caesar, 2019), which will compromise the intestinal barrier. This allows endotoxins to enter the circulation and activate immune responses, thereby mediating inflammatory reactions (Mohr et al., 2022; Di Lorenzo et al., 2019; Candelli et al., 2021). These reactions include releasing key pro-inflammatory cytokines, activating genes involved in inflammation and immune responses, that decrease sperm motility (O'Doherty et al., 2016; Parker and Palladino, 2017). For example, LPS from *Escherichia coli* can stimulate immune responses in healthy male mice, leading to the production of pro-inflammatory cytokines such as IL-17A, mediating immune and inflammatory responses in testicular tissue. This results in widespread necrosis of testicular parenchyma, damage to the epithelial cells of seminiferous tubules, reduction in testosterone levels within the testes, ultimately impairing testicular tissue, decreasing sperm production, reducing motility, and enhancing DNA fragmentation (Khanmohammad et al., 2021; Folliero et al., 2022; Li et al., 2024). Additionally, LPS-induced epididymitis in rats exhibits leukocyte infiltration and fibrosis in the caudal epididymis, downregulating the expression of rat-specific *β*-defensin SPAG11E, disrupting SPAG11E binding with sperm, damaging blood-epididymal barrier permeability, and sperm viability (Cao et al., 2010; Wang et al., 2019). Research by Brecchia G and others (Brecchia et al., 2010; Collodel et al., 2012) demonstrates that LPS-mediated subacute inflammation can disrupt rabbit testicular structure and sperm membrane integrity. After 30 days of LPS exposure, rabbit sperm membrane integrity and the number of necrotic sperm are severely affected, peaking at the end of the 56-day spermatogenic cycle. Supplementation with testicular vitamin K may help inhibit inflammatory signal transduction and improve LPS-induced reduction in testicular testosterone synthesis, maintaining stable testosterone levels (Takumi et al., 2011).

### 2.2 Oxidative stress and antioxidant protection

Sperm are susceptible to oxidative stress (OS), which refers to the imbalance between the generation of reactive oxygen species (ROS) and the cellular antioxidant defense systems (Barati et al., 2020). Spermiogenesis involves an oxidative process that requires controlled levels of ROS to trigger phosphorylation. Thus, at physiological concentrations, ROS are essential for normal sperm function, playing critical roles in sperm maturation, capacitation, hyperactivation, and acrosome reaction processes. However, excessive ROS can lead to OS, causing structural and functional damage to sperm cells, manifested as impaired energy metabolism, protein oxidation, lipid peroxidation, and DNA damage, ultimately resulting in reduced sperm motility and viability (Du Plessis et al., 2015; Aitken, 2017).

The GM can influence the host’s antioxidant defense system, thereby affecting sperm production and motility (Uchiyama et al., 2022; Magill and MacDonald, 2023). Antioxidant enzymes such as superoxide dismutase (SOD), glutathione peroxidase (GPX), peroxiredoxin (PRDX), thioredoxin, and glutathione-S-transferase exhibit antioxidant activity, neutralizing free radicals and other oxidative stressors to reduce oxidative damage. Certain probiotics or specific bacterial strains can produce antioxidants like glutathione and superoxide dismutase, which are essential for generating healthy sperm, maintaining sperm quality to ensure vitality, energy acquisition, and DNA integrity, thereby protecting sperm from oxidative harm (O'Flaherty and Scarlata, 2022; Oliveira et al., 2024; Wang et al., 2017). Studies indicate that PRDX regulates ROS levels, preventing oxidative stress during human sperm maturation processes (Lee et al., 2017) Further research by Fernandez MC (Fernandez and O'Flaherty, 2018; Fernandez et al., 2019) and others has highlighted peroxiredoxin 6 as a key antioxidant enzyme maintaining human sperm vitality and DNA integrity. Peroxiredoxin 6 regulates the phosphoinositide 3-kinase (PI3K) /protein kinase B (AKT) pathway to eliminate excessive ROS and maintain sperm vitality, thereby preventing oxidative damage.

Supplementation with antioxidants such as vitamins E and C, selenium, glutathione, coenzyme Q10, carotenoids, and l-carnitine can modulate GM, reducing sperm damage induced by oxidative stress (Beygi et al., 2021; Li et al., 2023). For instance, selenium (Se), a renowned antioxidant, significantly influences gut microbial composition, male sperm quality, and fertility. Research indicates associations between selenium binding protein (SeAlb), Escherichia/Shigella species, and glutathione peroxidase (GPx) (Rayman, 2012; Ramírez-Acosta et al., 2022). Studies by Sun et al. (2023) and Zeng et al. (2024), and others have shown that selenium gluconate (SeGlu) derivatives, novel organic selenium compounds, reduce the abundance of detrimental bacteria such as Rikenella, Barnesiella, Tenacibaculum, Acinetobacter, Bacteroides, and Alistipes, while increasing beneficial microbes like Intestinimonas, Christensenella, Coprococcus, Butyrivibrio, Clostridium, Ruminococcus, Lactobacillus, and Lactococcus. This supplementation enhances rat sperm quality by reducing harmful bacterial colonization, modulating GM, and decreasing sperm damage induced by oxidative stress.

Furthermore, dysbiosis of GM increases oxidative stress within the host, making cells more susceptible to oxidative damage, triggering immune responses, inflammation, and other pathological changes (Ferro et al., 2020), impairing sperm production and function. Dysbiosis-induced LPS induce oxidative stress-mediated mitochondrial damage in sperm, leading to significant mitochondrial ultrastructural changes and increased mitochondrial reactive oxygen species. This abnormal activation of oxidative phosphorylation (OXPHOS) and mitochondrial membrane lipid peroxidation result in sperm oxidative damage, reducing boar sperm motility and vitality (He et al., 2017). Research has shown that glyphosate (GLY) -induced dysbiosis of GM increases local interleukin (IL) -17A production (Liu et al., 2021), subsequently activating testicular oxidative damage, manifesting as impaired testicular structure, decreased sperm vitality, and increased sperm deformity rates.

### 2.3 Metabolites of GM

**Figure 1:**
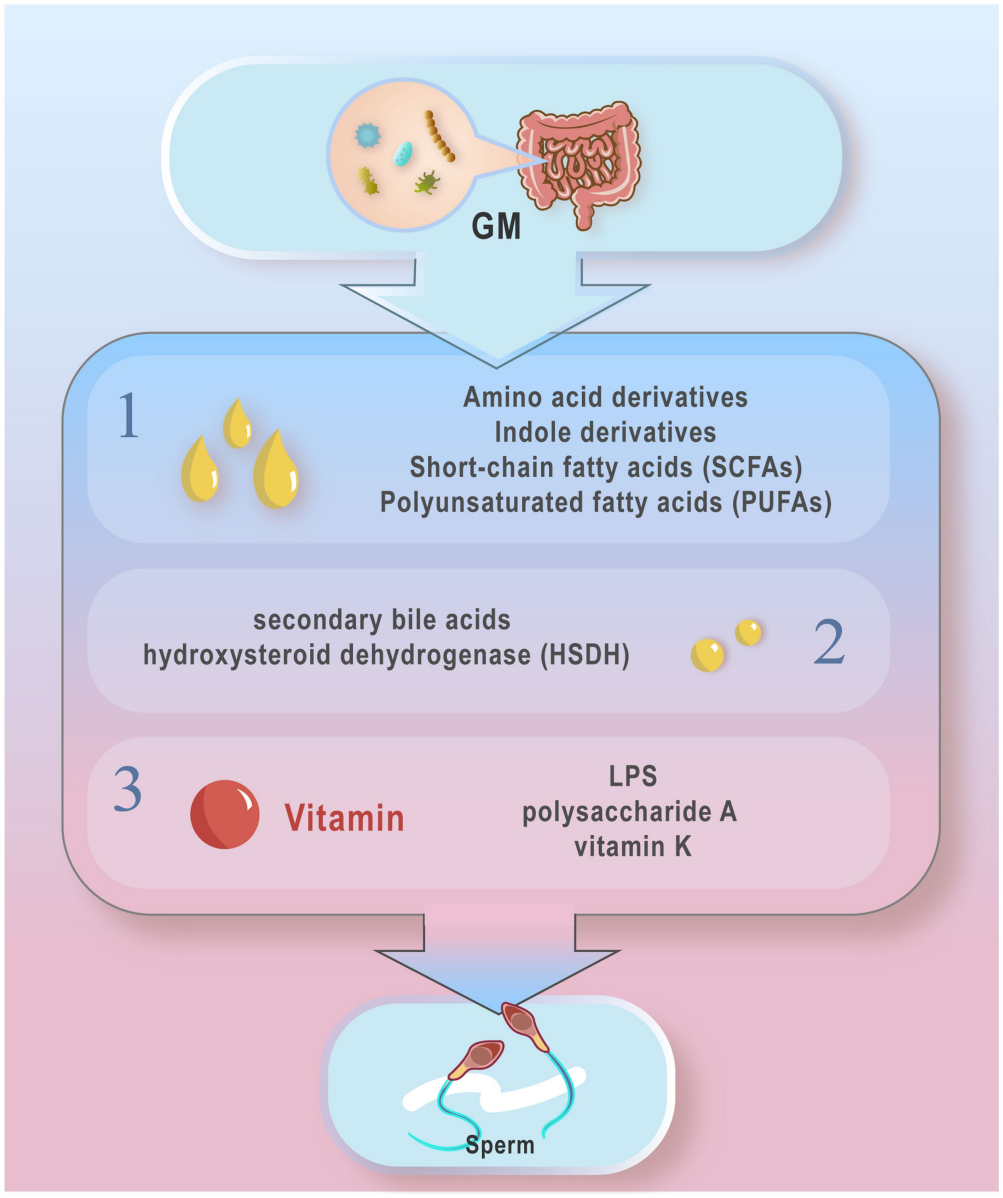
Typical gut microbiota metabolites from different sources. According to different sources, these metabolites can be divided into three main types: 1. Metabolites directly produced by the gut microbiota from the diet: short chain fatty acids (SCFAs), polyunsaturated fatty acids (PUFAs) amino acid derivatives, and indole derivatives; 2. Metabolites produced by the host and modified by the gut micro- biota, secondary bile acids and hydroxysteroid dehydrogenases (HSDH); 3 Metabolites synthesized from novel LPS, polysaccharide A, and vitamin K, etc. The metabolites of GM may influence the host’s Spermatogenesis and motility.

The influence of GM metabolites on host health extends to semen quality. GM produce a diverse array of metabolites with varied biological activities. These metabolites can be categorized into three main types based on their origins (Figure 1): Metabolites directly synthesized by GM from dietary sources, including short-chain fatty acids (SCFAs), polyunsaturated fatty acids (PUFAs), and amino acid derivatives; Metabolites initially produced by the host and subsequently modified by GM, such as secondary bile acids and hydroxysteroid dehydrogenase (HSDH); Metabolites synthesized *de novo*, such as LPS and vitamin K (Liu et al., 2022a; Lv et al., 2024). Alterations in GM composition can impact the levels of these metabolites, consequently influencing sperm production and quality (Wang et al., 2023a). For instance, decreased levels of *RuminococcaceeNK4A214_group* in the gut correlate with reduced bile acid levels, impairing spermatogenesis and decreasing spermatogenic cell counts (Zhang et al., 2021). Moreover, the GM-derived metabolite 3-hydroxyphenylacetic acid (3-HPAA) has been shown to inhibit ferroptosis-mediated mechanisms and promote spermatogenesis in aging mice (Jin et al., 2023). Supplementation with dietary fiber enhances GM composition in boars, stimulating the production of SCFAs and thereby improving sperm production and semen quality (Lin et al., 2022). This review focuses on the impact of key GM metabolites such as SCFAs, secondary bile acids, tryptophan and indole derivatives, and vitamins on sperm health.

SCFAs are metabolites produced by GM, particularly probiotics and *Bacillus subtilis*, through the fermentation of cellulose and unabsorbed carbohydrates. They play a crucial role in regulating gut homeostasis and influencing health and disease outcomes (van der Hee and Wells, 2021; Fusco et al., 2023). SCFAs are involved in regulating sperm production and motility; for instance, dietary supplementation with sodium butyrate (SB) in roosters has been shown to enhance semen volume, sperm motility, sperm concentration, and reduce abnormal sperm percentages. Additionally, it enhances the enzyme activity of GPx and SOD in adult roosters at 45 weeks, promoting testosterone secretion and testicular growth (Alhaj et al., 2018). SCFAs can improve intestinal microbiota altered by a high-fat diet (HFD), regulate lipid metabolism to enhance spermatogenesis, and improve semen volume and fertility by producing n-3 polyunsaturated fatty acids (Hao et al., 2022). They also increase beneficial enterobacteria, reduce harmful bacteria, elevate levels of acetic acid and butyric acid in feces, and enhance blood levels of testosterone, DHA, EPA, promoting spermatogenesis, and improving sperm concentration and vitality in type 2 diabetes (Yan et al., 2022; Zhou et al., 2023).

Secondary bile acids are a type of bile acid formed after metabolism by GM. They significantly influence host metabolism and immune response by modulating bile acid pool circulation and overall fat metabolism (Fogelson et al., 2023). Altering the GM structure affects bile acid metabolism, which in turn influences host metabolism and immune response (Tian et al., 2020; Lee et al., 2024). Bile acids may impact sperm production and quality through their regulatory effects on host metabolism and immune response. Research indicates that heat stress-induced dysbiosis of GM impairs spermatogenesis by altering secondary bile acid metabolism in the gut (He et al., 2024). Moreover, *Aspergillus fumigatus* regulates secondary bile acid metabolism by promoting colonization of bile salt hydrolase (BSH) metabolizing bacteria, thereby enhancing retinol absorption in the host gut and improving testicular retinoid levels, which further improves spermatogenesis. Zhang et al. (2022) found that reduced levels of *RuminococcaceeNK4A214_group* lead to decreased bile acid levels, causing abnormal vitamin A metabolism in the intestine and resulting in abnormal sperm.

Tryptophan is an amino acid metabolized into indole, a primary product of tryptophan metabolism. In the intestine, GM further metabolizes indole into various derivatives such as indole-3-propionic acid (IPA) and 3-hydroxyindole, which significantly influence host health, disease, and aging (Wang et al., 2024a; Gupta et al., 2023). IPA inhibits GM dysbiosis and intestinal endotoxin leakage (Zhao et al., 2019). Indole-derived metabolites upregulate CatSper protein expression, enhance testosterone secretion, and increase StAR protein expression to mitigate testicular injury induced by Cisplatin (II), inhibit OS and inflammation, and restore sex hormone levels (Afsar et al., 2022). The potential effects of tryptophan and its derivatives on sperm warrant further investigation.

### 2.4 Epigenetic modifications induced by GM and their impact on host physiology

The GM and epigenetic processes are dynamic and influenced by environmental factors and diet (Li et al., 2022c). Epigenetic modifications refer to chemical alterations of certain parts of the genome that do not involve changes in the DNA sequence itself. These modifications alter the structure or modification status of DNA and its associated proteins, thereby regulating gene expression levels and functions. They include DNA methylation, histone modifications, chromatin remodeling, and modifications mediated by non-coding RNAs (Skvortsova et al., 2018; Xavier et al., 2019).

Epigenetic regulation is considered an effective mechanism by which the GM influences host physiological functions (Wang et al., 2024b). GM metabolites can induce epigenetic modifications, such as changes in DNA methylation and micro-RNA expression. Studies have shown that gut microbes like lactobacilli and bifidobacteria can influence DNA methylation by affecting the bioavailability of folate they produce (Ashonibare et al., 2024). Kumar et al. (2014) demonstrated that GM dominated by Firmicutes or Bacteroidetes correlates with differences in the methylation status of gene promoters associated with cardiovascular disease.

The GM may influence sperm genetic quality and offspring health through effects on host gene expression and epigenetic modifications. These effects can manifest in various ways, including changes in DNA methylation patterns or regulation of histone modifications, thereby impacting genetic stability and phenotypic characteristics of sperm (Woo and Alenghat, 2022; Ashonibare et al., 2024). For instance Liu et al. (2022b) found that water extracts of black tea alter tissue gene expression through GM modulation, changing the levels of major epigenetic modifications (DNA methylation) and regulating imprinting genes’ DNA methylation in sperm of high-fat diet-fed mice. The impact of GM on sperm via epigenetic modifications is evident but requires further investigation for clarification.

### 2.5 GM’s role in regulating sex hormones and spermatogenesis

The GM influences the host’s endocrine system, including regulation of sex hormones by affecting the hypothalamic–pituitary-gonadal (HPG) axis (Wang and Xie, 2022). Sex hormones are crucial for spermatogenesis and sperm activity, and dysbiosis of the GM may lead to abnormal changes in hormone levels, affecting sperm quality and quantity. Within the HPG axis, the hypothalamus coordinates the pulsatile release of gonadotropin-releasing hormone (GnRH), activating the pituitary-gonadal axis. GnRH stimulates the pituitary gland to produce luteinizing hormone (LH) and follicle-stimulating hormone (FSH), which are vital for male reproductive processes. LH regulates Leydig cell function and testosterone secretion, while FSH promotes germ cell division and sperm production, supporting the energy metabolism of testicular germ cells (Kaprara and Huhtaniemi, 2018).

The GM can modulate hormone levels through various pathways (He et al., 2021). Studies by Ashonibare (Ashonibare et al., 2024) suggest that the GM can directly influence the synthesis of hormone-related enzymes and participate in the enterohepatic circulation of hormones, thereby affecting the hypothalamic–pituitary-testicular (HPT) axis. Research by Shin JH (Shin et al., 2019) indicates that men with higher testosterone levels have a more diverse gut microbial community compared to others, with abundances of Bacteroides, Dorea, Ruminococcus, and Clostridium significantly correlating with testosterone levels. Similarly, research by Yan et al. (2024) shows that within the male GM, species like Coprobacter, Ruminococcus2, Barnesiella, Actinomyces, and Bifidobacterium are negatively correlated with sex hormone-binding globulin (SHBG) levels, whereas *α*-Proteobacteria are positively correlated.

The GM may be a primary regulatory factor in testosterone production and metabolism. Deng C (Li et al., 2024) and others propose interactions between testosterone and the GM, suggesting testosterone may regulate spermatogenesis through the blood-testis barrier (BTB). Tang (Tang et al., 2024) further supports Deng’s findings, showing that viscumin affects the immune microenvironment of the testes, downregulating serum testosterone levels in male mice by inhibiting Akkermansia, disrupting guanosine metabolism. Supplementation of guanosine restores testosterone secretion by repairing the BTB and serum lipopolysaccharide levels. *Clostridium scindens* American Type Culture Collection 35,704 converts primary bile acids into toxic secondary bile acids and converts glucocorticoids into testosterone by side-chain cleavage (Ridlon et al., 2013). Adolescent Bifidobacterium strains with 20β-HSDH activity can alter glucocorticoid metabolism in the gut, potentially serving as probiotics for testosterone-dependent diseases (Doden et al., 2019). Poutahidis et al. (2014) and colleagues demonstrate that male mice fed purified Lactobacillus have larger testes and higher serum testosterone levels compared to controls. Moreover, feeding mice with *Lactobacillus reuteri* significantly increases testosterone levels after 5 months, with significant enhancement in seminiferous tubule cross-sectional profiles and interstitial cell proliferation in the testes.

Furthermore, the GM can regulate the permeability of the BTB, influencing hormone levels and thereby modulating sperm production and motility. The BTB is a critical ultrastructure in the testes supporting meiosis and post-meiotic spermatogenic cell development (Cheng and Mruk, 2012). Dysbiosis of the GM can increase inflammation, regulating oxidative stress-related enzyme activity, testosterone levels, and BTB permeability (Guo et al., 2024). Al-Asmakh et al. (2014) and others demonstrate that the microbiota regulates BTB permeability through modulation of intercellular adhesion, secreting high levels of butyrate, which restores BTB integrity in germ-free (GF) mice and normalizes levels of cell adhesion proteins, with intercellular adhesion molecules (ICAMs) being critical regulatory molecules for spermatogenesis (Xiao et al., 2013).

## 3 Intervention methods: correcting dysbiosis of GM

### 3.1 Prebiotics, probiotics, and synbiotics

**Figure 2:**
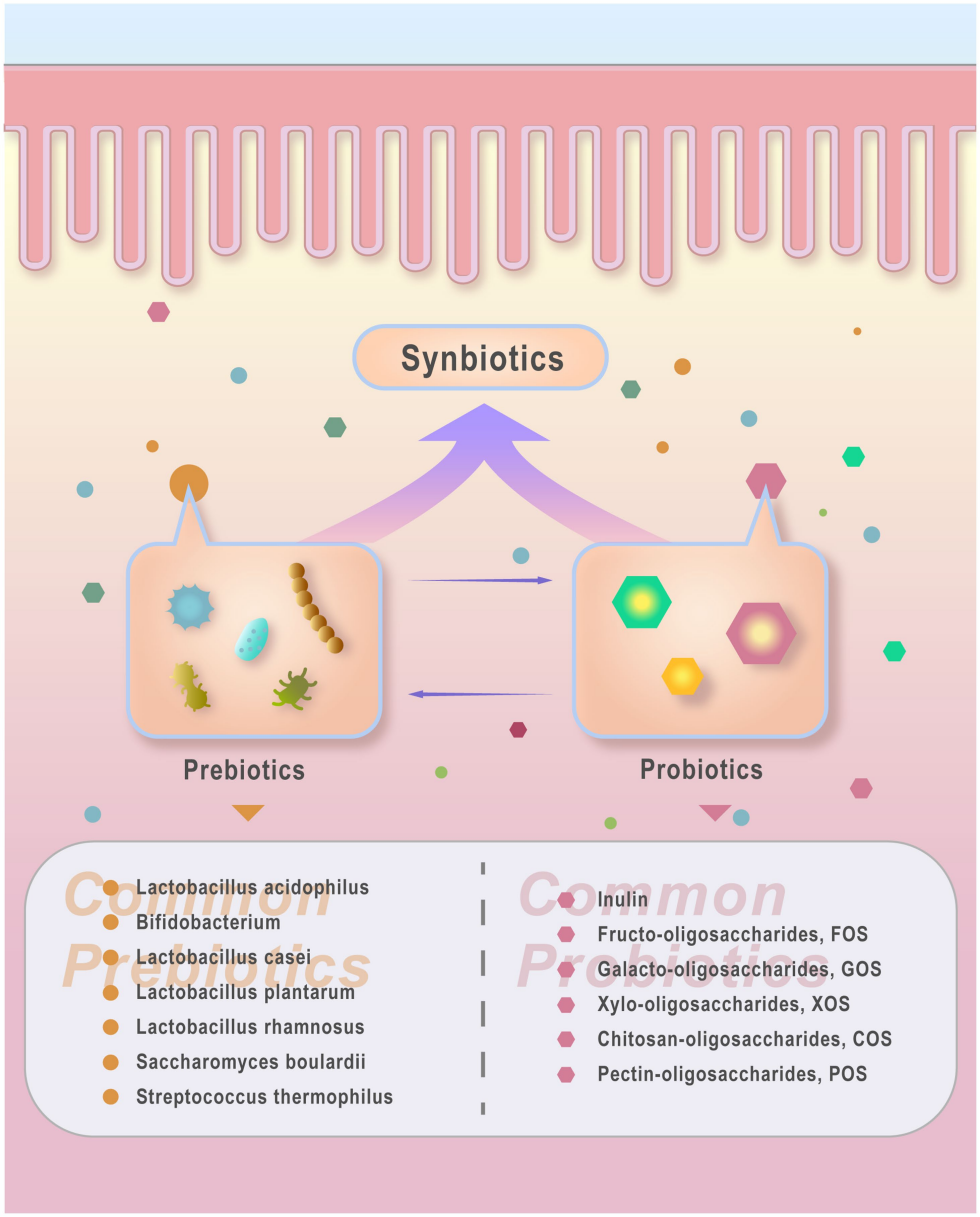
probiotics, prebiotics and synbiotics. Probiotics refer to beneficial live microorganisms that improve host health conditions. Common types include Lacto- bacillus acidophilus, Bifidobacterium, *Lactobacillus casei*, *Lactobacillus plantarum*, *Lactobacillus rhamnosus*, and yeast such as Saccharomyces boulardii. Other probiotics like *Streptococcus thermophilus* are also recognized for their beneficial effects on host health. Prebiotics refer to food components that cannot be digested or absorbed by the host but can be utilized by beneficial gut bacteria, thereby promoting the growth or activity of probiotics. Common prebiotics include Inulin, fructo-oligosaccharides (FOS), galacto-oligosaccharides (GOS), xylo-oligosac- chandes (XOS), chitosan-oligosaccharides (COS), and pectin-oligosaccharides (POS). Synbiotics refer to products combining probiotics and prebiotics that coexist and interact synergistically. Synbiotics contribute to enhancing gut microbiota by delivering probiotics (live beneficial microorganisms) and prebiotics (compounds that foster probiotic growth).

Prebiotics refer to specific non-digestible food components beneficial to humans; Probiotics are live microorganisms in the gut; and synbiotics is composed of a mixture of prebiotics and probiotics. Prebiotics can stimulate the growth and activity of beneficial gut flora to improve host health. Probiotics confer health benefits to the host by colonizing the intestinal tract, rebalancing GM, and inhibiting the growth of harmful bacteria. Synbiotics offer a broader and more comprehensive probiotic effect through synergistic interactions of multiple strains (Ashonibare et al., 2024; Swanson et al., 2020; Gibson et al., 2017; Hill et al., 2014).Prebiotics, probiotics, and synbiotics have the potential to rectify dysbiosis of GM, influencing various host functions through colonization, pathogen eradication, and induction of host cell responses, thereby serving as microbial management tools to enhance host health (Sanders et al., 2019; Yadav et al., 2022) (Figure 2).

Supplementation with prebiotics, probiotics, and synbiotics can ameliorate OS and inflammation, adjust sex hormone levels, thereby improving sperm quality. Reshaping of GM following probiotic supplementation reduces proliferation of pathogenic bacteria, enhances intestinal barrier function, decreases oxidative stress, restores balance of SCFAs, and improves testicular function by repairing seminiferous tubule structure and increasing spermatogonial stem cells (Wu et al., 2024b), while also reducing gut-derived inflammatory mediators circulating in the bloodstream (Cai et al., 2023). For instance, supplementation with *Lactobacillus rhamnosus NCDC-610* [and *Lactobacillus fermentum NCDC-400* with prebiotics such as fructooligosaccharides (FOS)] enhances activities of catalase and superoxide dismutase, IL-6, IL-10, and tumor necrosis factor-alpha (TNF-*α*), thereby improving oxidative stress and inflammation, mitigating sperm defects induced by restraint stress, and enhancing gut health (Akram et al., 2023). Studies by Akram et al. (2022), Dardmeh et al. (2017), and others similarly demonstrate that supplementation with *Lactobacillus fermentum NCDC 400* and *Lactobacillus rhamnosus NCDC 610*, *Lactobacillus rhamnosus PB01*, along with FOS, can reduce OS damage, maintain testosterone concentrations, restore testicular structure, and improve sperm vitality and motility parameters in diet-induced obesity models.

Research indicates that synbiotics (*Lactobacillus paracasei* + arabinoxylan oligosaccharides + FOS + L-glutamine) can regulate FSH, LH, and testosterone levels in idiopathic oligoasthenoteratozoospermia patients and improve semen volume and sperm quality/quantity (Maretti and Cavallini, 2017). Khan et al. (2024) and others have also shown that supplementation with *Lactobacillus rhamnosus*, Bifidobacterium, and galactooligosaccharides can enhance immature male Japanese quail estrogen, testosterone, FSH, and LH steroid hormone receptor expression through GM modulation, increase catalase to improve oxidative stress, promote testicular weight, and gonadosomatic index (GSI). FamiLact (probiotics + prebiotics) can alleviate oxidative stress, improve sperm concentration, vitality, and abnormal morphology, and reduce sperm DNA damage (Abbasi et al., 2021). Further research by Mahiddine et al. (2023) indicates that supplementation with *Lactobacillus rhamnosus* for 6 weeks increases relative abundance of Actinobacteria, Bacillus, and Streptomyces while decreasing Clostridium and Enterococcus, thereby enhancing sperm kinetic parameters, vitality, and acrosome integrity, and upregulating mRNA levels of genes associated with DNA repair and antioxidation.

### 3.2 Fecal microbiota transplantation

Fecal microbiota transplantation (FMT) involves transferring GM derived from healthy donor feces into the gastrointestinal tract of patients to treat dysbiosis-related diseases by altering GM composition. The efficacy of FMT may be linked to the specific implantation of donor phages (Wang et al., 2022; Liu et al., 2023). Increasingly valued and recognized as a novel treatment method to enhance semen quality, FMT has gained attention (Hao et al., 2022).

FMT has shown potential to mitigate inflammation and improve testicular diseases in male mice induced by GM dysbiosis from microplastics (MPs), thereby enhancing semen quality (Zhang et al., 2023; Wen et al., 2022). It can also alleviate male obesity and fertility decline caused by a HFD by enhancing systemic and testicular metabolism. For instance, studies by Hao et al. (2022), Hao et al. (2022), Yan et al. (2022) and others demonstrated that modifying GM through FMT combined with alginate oligosaccharides (AOS) (A10-FMT) improved reduced semen quality (sperm concentration and vitality) caused by a high-fat diet. A10-FMT enhanced blood metabolism and increased beneficial GM such as lactobacilli and allobacilli, including small intestinal lactobacilli, thereby elevating blood and/or testicular levels of butyric acid, docosahexaenoic acid (DHA), eicosapentaenoic acid (EPA), and testosterone, promoting spermatogenesis, and thereby improving sperm concentration, vitality, and semen quality affected by type 1 (T1D) and type 2 diabetes (T2D) through the gut-microbiota-testis axis (Hao et al., 2022).

Given the limitations in acceptance and reproductive feasibility of fecal transplantation in clinical practice, researchers have explored alternative approaches for FMT, such as transplanting viral groups, bacterial communities (e.g., phage transplantation), and fungal groups (e.g., Candida genus). Future advancements in FMT are anticipated to focus more on transplanting specific components of fecal microbiota, such as bacterial or viral components (Lam et al., 2022; Wu et al., 2023; Yu et al., 2023). Consequently, future developments like fecal bacteriophage transplantation (FBT) and fecal virome transplantation (FVT) offer potential avenues to modulate GM to enhance sperm production and motility.

### 3.3 Traditional natural herbal extracts

Traditional natural herbs have been widely used in clinical treatment and health care in many countries and regions (Jia et al., 2022) The GM and traditional natural herbs can interact synergistically, with herbs capable of modulating GM composition (An et al., 2019). They enhance sperm production and motility through mechanisms such as elevating SCFA levels, regulating bile acid metabolism, reducing trimethylamine oxide production, and mitigating inflammatory factor release (Li et al., 2021).

Ginseng, widely used in clinical settings, is noted for its energizing effects and fatigue-reducing properties. Research indicates that ginsenosides (Zhang et al., 2024) significantly enhance bile acid enterohepatic circulation via the FXR/CYP7A1 pathway, restore GM diversity, rebalance the Firmicutes/Bacteroidetes ratio, and ameliorate sperm damage and density (Ji et al., 2024). Chestnut polysaccharides (CPs) improve the testicular microenvironment, notably increasing germ cell counts in seminiferous tubules, adjusting GM composition by enriching Firmicutes, Proteobacteria, Bacteroidetes, Actinobacteria, and other phyla. Studies suggest that CPs metabolize through steroid hormone biosynthesis to enhance sperm production (Sun et al., 2022; Yu et al., 2020). *Rhodiola rosea* glycoside (Wang et al., 2023b; Wang et al., 2024c) inhibits LPS entry into the circulatory system, activates SCFA receptor mRNA expression, fortifies the intestinal barrier, alleviates orchitis, and enhances semen quality via GM regulation and metabolite adjustment. *Cornus officinalis* glycoside alleviates diabetes-induced testicular injury by inhibiting the AGEs-RAGE-p38 MAPK pathway, modulates intestinal flora, markedly reverses flora distribution, increases testosterone, LH, and FSH levels, and improves sperm count and vitality (Liu et al., 2021; Chen et al., 2016).

Cordyceps militaris, a parasitic fungus with medicinal properties, is utilized in food and medicine. Cordyceps polysaccharides (SeCMP) extracts exhibit structural diversity (Wu et al., 2024a) and repair intestinal mucosal damage from LPS. By augmenting lactobacilli abundance while reducing Akkermansia and Bacteroidetes, SeCMP mitigates intestinal microbiota imbalance (Wu et al., 2022). SeCMP corrects metabolic disorders, enhances testosterone synthesis in mice, raises androgen levels, increases seminiferous tubule area, thereby boosting sperm concentration and vitality in mice (Lin et al., 2022; Lin et al., 2007; Huang et al., 2023). Furthermore, SeCMP decreases rumen cocci abundance in infertile male rats, increases Romboutsia abundance, lowers serum LPS levels, and enhances sperm production by restoring intestinal microbiota diversity and inhibiting epididymitis in infertile male rats (Sheng et al., 2023).Traditional natural herbal resources are abundant and can effectively regulate GM and sperm quality. There is great potential to improve sperm quality by regulating GM, with further exploration needed regarding its application value.

In addition to the aforementioned measures, improving male fertility through gut microbiota regulation remains an ongoing area of research. Modifying lifestyle habits could potentially enhance gut microbiota, restore its balance, and improve semen quality. Lin et al. have demonstrated that dietary fiber supplements can positively affect gut microbiota and boost SCFA production, which in turn improves sperm production and semen quality (Lin et al., 2022). Conversely, chronic alcohol consumption can disrupt gut microbiota, leading to metabolic disorders, increased serum endotoxins and inflammatory cytokines, orchitis, abnormal gene expression, and ultimately, reduced sperm quality (Liu et al., 2022b).

## 4 Conclusion and outlook

**Figure 3:**
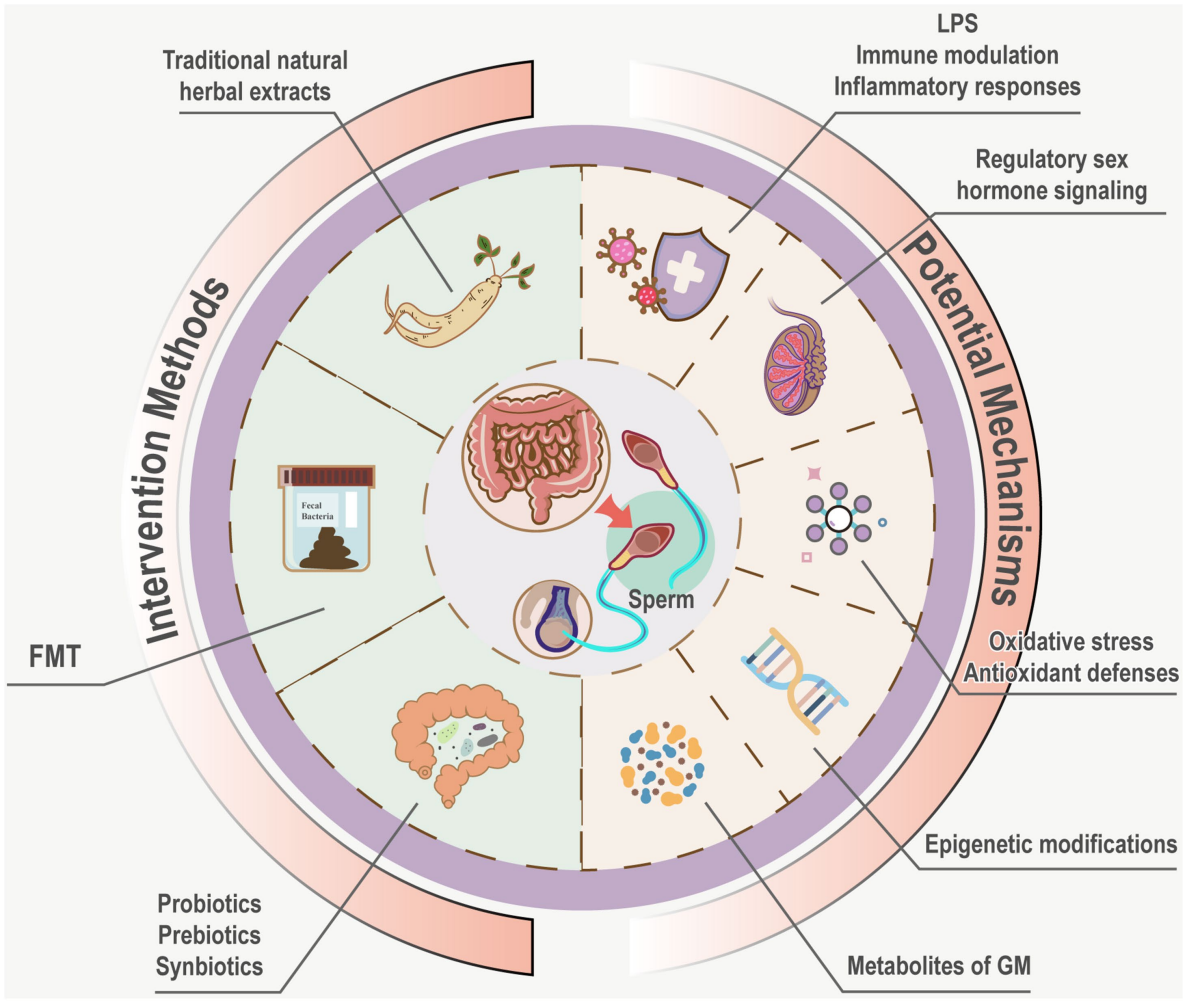
Potential mechanisms and intervention measures of GM influence sperm. Potential mechanisms of GM on sperm encompass immune modulation, inflammatory responses mediated by endo- toxins, oxidative stress, antioxidant defenses, metabolites of GM, epigenetic modifications, regulatory sex hormone signaling Interventions such as probiotics, prebiotics, synbiotics, FMT, and Traditional natural herbal extracts are hypothesized to rectify dysbiosis, offering avenues to modulate gut microbiota and enhance spermatogenesis and motility.

The GM exerts dual effects on sperm through endotoxin-mediated immune and inflammatory responses, oxidative stress and antioxidant protection, metabolites of GM, epigenetic modifications, regulatory sex hormones. Prebiotics, probiotics, symbiotics, fecal microbiota transplantation, and Traditional natural herbal extracts offer potential for rectifying dysbiosis in the GM and regulating spermatogenesis and motility (Figure 3). Due to the unclear mechanisms through which specific GM and their metabolites influence sperm quality, methods aimed at enhancing male fertility by modulating the GM remain experimental, and clinical evidence is still needed. Future research should investigate the specific effects of particular GM and their metabolites on sperm quality, as well as explore the regulatory and mechanistic roles of different prebiotics, probiotics, and traditional medicines on GM and sperm quality. Given the influence of GM on sperm, continued focus on the Testis-Gut microbiota Axis is warranted, emphasizing interconnections and mutual impacts in future research directions. The diversity of GM species and the complexity of their mechanisms underscore the extensive journey ahead in this field.
